# Maternal exposure to childhood maltreatment and mental and behavioral disorders in children

**DOI:** 10.1007/s00787-022-02090-8

**Published:** 2022-10-01

**Authors:** Aino Airikka, Marius Lahti-Pulkkinen, Soile Tuovinen, Kati Heinonen, Jari Lahti, Polina Girchenko, Anna Lähdepuro, Riikka Pyhälä, Darina Czamara, Pia Villa, Hannele Laivuori, Eero Kajantie, Elisabeth B. Binder, Katri Räikkönen

**Affiliations:** 1 Department of Psychology and Logopedics, Faculty of Medicine, University of Helsinki, Haartmaninkatu 3, P.O. Box 21, 00014 Helsinki, Finland; 2 The Finnish Institute for Health and Welfare, Helsinki, Finland; 3 University of Edinburgh, Edinburgh, UK; 4 Welfare Sciences, Faculty of Social Sciences, University of Tampere, Tampere, Finland; 5 Department of Translational Research in Psychiatry, Max Planck Institute of Psychiatry, Munich, Germany; 6 Obstetrics and Gynecology, University of Helsinki and Helsinki University Hospital, Helsinki, Finland; 7 Medical and Clinical Genetics, University of Helsinki and Helsinki University Hospital, Institute for Molecular Medicine Finland (FIMM), Helsinki, Finland; 8 Helsinki Institute of Life Science, University of Helsinki, Helsinki, Finland; 9 Department of Obstetrics and Gynecology, Center for Child, Adolescent and Maternal Health Research, Tampere University Hospital and Faculty of Medicine and Health Technology, Tampere University, Tampere, Finland; 10 PEDEGO Research Unit, MRC Oulu, Oulu University Hospital and University of Oulu, Oulu, Finland; 11 Children’s Hospital, Helsinki University Hospital and University of Helsinki, Helsinki, Finland; 12 Department of Clinical and Molecular Medicine, Norwegian University for Science and Technology, Trondheim, Norway

**Keywords:** Childhood maltreatment, Psychopathology, Intergenerational, Cohort study

## Abstract

Exposure to maltreatment in childhood is associated with lifelong risk of mental and behavioral disorders. Whether the effects extend to the next generation remains unclear. We examined whether maternal exposure to childhood abuse and neglect in her own childhood were associated with mental and behavioral disorders and psychiatric symptoms in her children, and whether maternal lifetime mental and behavioral disorders or lower education level mediated or added to the effects. Mothers (*n* = 2252) of the Prediction and Prevention of Preeclampsia and Intrauterine Growth Restriction cohort study completed the Childhood Trauma Questionnaire and reported on their education and their 7.0–12.1-year-old children’s psychiatric symptoms using the Strengths and Difficulties Questionnaire. We identified lifetime mental and behavioral disorder diagnoses for the mothers and diagnoses for their children from birth (2006–2010) until 8.4–12.8 years (12/31/2018) from Care Register for Health Care. We found that maternal exposure to childhood abuse, but not neglect, was associated with higher hazards of mental and behavioral disorders (hazard ratio 1.20, 95% confidence interval 1.06–1.37) in children. These associations were partially mediated by maternal mental and behavioral disorders and education (proportion of effect size mediated: 23.8% and 15.1%, respectively), which together with maternal exposure to childhood abuse added to the hazard of mental and behavioral disorders in children. Similar associations were found for maternal exposure to childhood abuse and neglect with psychiatric symptoms in children. To conclude, maternal exposure to childhood maltreatment is associated with mental and behavioral disorders and psychiatric symptoms in children. Our findings call for interventions to prevent intergenerational transmission.

**Supplementary Information:**

The online version contains supplementary material available at 10.1007/s00787-022-02090-8.

## Introduction

Childhood maltreatment, including physical, emotional and sexual abuse, and physical and emotional neglect [1], is common affecting 13–36% of children [2]. There is extensive evidence of adverse health impacts of childhood maltreatment, and children exposed to childhood maltreatment have an increased risk of mental and behavioral disorders across the lifespan [3–9]. Increased risks are present for various mental and behavioral disorders, including anxiety and depressive disorders [3, 4, 8], substance use disorders [4, 8], eating disorders and childhood behavioral and conduct disorders [4], attention-deficit-hyperactivity-disorders [9], psychotic disorders [6], personality disorders [10], and perinatal anxiety and depressive disorders [5]. Meta-analytic evidence from prospective studies suggests that these associations may be causal [3, 4]. Furthermore, a recent Mendelian randomization study suggests a causal relationship of genetic variants associated with childhood maltreatment and depression, attention-deficit-hyperactivity-disorder, and schizophrenia [11].

Apart from the within-generation impacts, there is increasing evidence that the adverse health effects of exposure to childhood maltreatment may be transmitted into the next generation [7, 12–18]. Several studies show that children whose mothers had themselves been exposed to childhood maltreatment have an increased risk of psychiatric symptoms [7, 12–18]. However, previous studies have mostly relied on the mother as the informant of maternal and child phenotypes. Therefore, estimates may be affected by rater bias and shared method variance, potentially resulting in inflated parent-to-child associations [4, 17, 19]. Hence, despite the relatively well-established associations with psychiatric symptoms in children [12], it remains elusive whether the effects of maternal exposure to childhood maltreatment extend to more severe and objectively defined diagnosed mental and behavioral disorders in the next generation.

To our knowledge, only two small-scale studies on 198–203 mother–child dyads in one Australian cohort using a validated measure of childhood maltreatment have examined whether maternal exposure to childhood maltreatment is associated with the risk of reliably diagnosed mental and behavioral disorders in children [20, 21]. In these studies, maternal exposure to childhood maltreatment was associated with increased risks of emotional disorders in 4-year-old children [20, 21]. The limited existing evidence calls for further studies with larger sample sizes and including child diagnoses across the whole spectrum of childhood mental and behavioral disorders.

A related study question is whether the possible effects of maternal exposure to childhood maltreatment on mental and behavioral disorders in children are independent of maternal mental and behavioral disorders and/or whether these maternal disorders mediate or add to the effects of maternal exposure to childhood maltreatment. Maternal mental and behavioral disorders are themselves associated with maternal exposure to childhood maltreatment [3, 5] and constitute key risk factors for mental and behavioral disorders in children [22]. The same holds true for maternal socioeconomic disadvantage, which is associated with childhood maltreatment [23, 24] and with the risk of mental and behavioral disorders in children [25]. Maternal self-reported psychiatric symptoms [13, 15, 18] and physician-diagnosed major depressive disorder [16] have indeed been shown to at least partially mediate the effects of maternal exposure to childhood maltreatment on mother-rated psychiatric symptoms in children. However, we do not know of any studies examining whether such mediation effects are present on diagnosed mental and behavioral disorders in children. We are neither aware of studies examining maternal socioeconomic disadvantage as a possible mediator of the intergenerational associations or of studies examining the possible additive effects of these three types of maternal adversities.

To fill in these knowledge gaps, we examined in a large sample of 2252 Finnish mothers and their children, whether maternal exposure to abuse and/or neglect in her own childhood was associated with higher hazards of mental and behavioral disorders in her children, followed up from the child’s birth until the age of 8.4–12.8 years. We also examined whether these associations were independent of maternal lifetime mental and behavioral disorders and lower education level, or whether these factors mediated and/or added to the effects of maternal exposure to childhood maltreatment. Furthermore, to consolidate the previous evidence [12], we studied associations with psychiatric symptoms in children, rated by the mother at the child’s age of 7.0–12.1 years.

## Methods

### Participants

The Prediction and Prevention of Preeclampsia and Intrauterine Growth Restriction (PREDO) study originally comprised 4777 women and their singleton children born alive in Finland between 2006 and 2010 [26]. The PREDO cohort was originally set up to identify risk factors for preeclampsia and intrauterine growth restriction. The cohort comprises two study arms: the clinical and epidemiological study arms.

The women in the clinical arm included 1079 women with a known risk factor status for preeclampsia and/or intrauterine growth restriction. Women were not eligible to participate in the clinical study arm if they had asthma, allergy to aspirin, previous peptic ulcer, previous placental ablation, inflammatory bowel diseases, rheumatoid arthritis, haemophilia, or thrombophilia, or were smoking during pregnancy [26].

The epidemiological study arm included 3698 women who may or may not had had risk factors for the above-mentioned conditions. For the epidemiological arm, there were no eligibility criteria.

All women were recruited to the study at their first ultrasound screenings in early pregnancy at ten study hospitals in Southern and Eastern Finland. Three women have since withdrawn consent. In 2016, we invited 4505 (94.3% of the original sample with informed consent) mother–child dyads with available contact information to a follow-up at the child’s age of 7.0–12.1 years. Of them, 2323 (51.6%) dyads participated in the follow-up, of whom data on maternal childhood maltreatment exposure and child outcome variables were available for 2252 (97.1%) dyads.

Compared to the non-participants in the original cohort (*n* = 2522), the participating mothers (*n* = 2252) were older at childbirth (32.0 vs. 31.0 years, *p* < 0.001) and had higher education levels (tertiary 74.6% vs. 54.5%, *p* < 0.001). The participating mothers (13.7% vs. 21.1%, *p* < 0.001) and their children (10.1% vs. 12.2%, *p* = 0.02) had less frequently been diagnosed with mental and behavioral disorders than the non-participants.

### Measures

#### Maternal exposure to childhood abuse and childhood neglect

When their children were 7.0–12.1 years old, the women completed the Childhood Trauma Questionnaire (CTQ) [27], which is a validated tool to measure five dimensions of exposure to childhood maltreatment [28]. These dimensions include physical, emotional, and sexual abuse and physical and emotional neglect. The 28 CTQ items are rated on a five-point scale ranging from never true (1) to very often true (5). While the CTQ does not precisely specify the age at exposure to childhood maltreatment, in the general instructions of the Finnish CTQ version used in this study, the respondents are asked to choose the answer option that best describes their recollections of their own childhood. The specific questionnaire items then ask the respondent to describe whether the statements made in the 28 items held true when the respondent was growing up. There are five items assessing each type of childhood maltreatment, while three validity items do not load on any dimension [28].

We used both continuous sum scores and binary cut-off scores as indicators of maternal exposure to childhood abuse and to childhood neglect.

We calculated a continuous sum score of maternal exposure to childhood abuse by summing up the scores of the scales on physical, sexual, and emotional abuse. We also calculated a continuous sum score of maternal exposure to childhood neglect by summing up the scores of the scales on emotional and physical neglect. Higher scores reflect more severe exposure to abuse and more severe exposure to neglect in childhood, respectively.

For the dichotomous cut-off scores (none-or-minimal vs. moderate-to-severe maltreatment), the cutoffs (≥ 10 physical abuse, ≥ 13 emotional abuse, ≥ 8 sexual abuse, ≥ 10 physical neglect, and ≥ 15 emotional neglect) provided by the CTQ manual were used [27]. Based on these cutoffs, we calculated binary variables reflecting maternal exposure to any type of moderate-to-severe childhood abuse and any type of moderate-to-severe childhood neglect.

#### Child mental health: mental and behavioral disorders and psychiatric symptoms in children

We assessed mental health in children both categorically with diagnosed mental and behavioral disorders identified from a validated nationwide medical register and dimensionally with mother-rated psychiatric symptoms assessed with a validated questionnaire.

Mental and behavioral disorder diagnoses in children were identified from the Finnish Care Register for Health Care (HILMO) from birth between 2006 and 2010 to 12/31/2018, when the children were 8.4–12.8 years-of-age. The nationwide HILMO register includes primary and subsidiary diagnoses of all inpatient hospital visits in Finland and all outpatient visits in public specialized medical care in Finland. These diagnoses are coded using International Statistical Classification of Diseases and Related Health Conditions, 10th Revision (ICD-10). The HILMO is best validated for primary diagnoses [29], and in our sample, 97.8% of the children with mental and behavioral disorders had a mental and behavioral disorder as the primary diagnosis. The HILMO is a validated tool for research in general [29], and also specifically for certain childhood mental and behavioral disorder diagnoses, including attention-deficit-hyperactivity-disorder [30] and autism spectrum disorder [31]. We examined any mental and behavioral disorder (ICD-10: F00-F99) in children as our primary outcome.

When the children were 7.0–12.1 years old, the mothers rated their child’s psychiatric symptoms with the Strengths and Difficulties Questionnaire (SDQ) [32]. The 20 SDQ items on total difficulties are rated on a three-point scale ranging from not true (0) to certainly true (2), with items tapping emotional symptoms, conduct problems, hyperactivity/inattention, and peer problems. A higher score indicates more total difficulties. We used the total difficulties score (Cronbach’s *α* = 0.81 for internal consistency) as our secondary outcome indicating dimensional psychiatric symptoms in children.

#### Covariates

As covariates in all analyses, we used child’s sex and maternal age (years) at childbirth, derived from Finnish Medical Birth Register (MBR). When diagnosed mental and behavioral disorders were examined as the outcome, we also used child’s birth year derived from the MBR. With psychiatric symptoms in children as the outcome, we used child’s age (years) at the psychiatric symptom assessment.

#### Covariates, mediators, and additive factors

We examined maternal lifetime mental and behavioral disorders and education level both as covariates, mediators, and as additive factors. We identified maternal lifetime mental and behavioral disorders from the HILMO with ICD-8 codes 290–315 until 1986; ICD-9 codes 290–319 in 1987–1995; and ICD-10 codes F00–F99 from 1996 onwards. We used data extracted until the child’s diagnosis for the analysis with diagnosed mental and behavioral disorders as the outcome and until the 7–12-year follow-up for the analysis with psychiatric symptoms in children as the outcome.

The mothers self-reported their highest achieved education level at the 7–12-year follow-up. We categorized maternal education level into primary or secondary vs. tertiary. If these data from the 7–12-year follow-up were missing, we extracted mother-reported data on education level from earlier follow-ups during pregnancy and at the child’s age of 1.9–6.3-years.

Mothers also reported on their income level and occupational status at the 7–12-year follow-up. We categorized maternal income level into lower vs. higher (≤ 2000 vs > 2000 Euros per month) and occupational status into employed or self-employed vs. unemployed vs. other (including women who were staying at home with children, who were students, who were retired, or who answered the answer option “other”). Supplementary Table S1 available online shows that lower maternal education level, lower maternal income level, and maternal unemployment status were all strongly and significantly associated with each other. While we present sample characteristics and univariate associations for all these three socioeconomic indicators, we will focus in our main analyses on maternal education as a covariate, mediator, and additive factor, since our data on it are the most complete.

### Statistical analysis

Statistical analyses were conducted with IBM SPSS Statistics version 27, R version 4.1.2 and Stata MP version 16. We estimated the associations of maternal exposure to childhood abuse and childhood neglect with mental and behavioral disorders in children with Cox regression models. We first adjusted the models for child’s birth year, child sex, and maternal age at childbirth. We then made further adjustments for maternal mental and behavioral disorders and education level. Continuous maternal childhood abuse and neglect sum scores were rank-order-normalized according to Blom’s formula and standardized (*z* scores). Before applying the Cox models, we tested for time-dependent effects. The hazard ratios did not change across time (*p* values ≥ 0.32).

We also studied if maternal mental and behavioral disorders and/or education level mediated the associations of maternal exposure to childhood abuse and/or neglect with mental and behavioral disorders in children. Mediation was tested with structural equation models and only if the criteria for mediation were met. These criteria included that (1) the independent variable was significantly associated with the mediator; (2) the independent variable was associated with the dependent variable; (3) the mediator was independently associated with the dependent variable; and (4) the effect size of the independent variable on the dependent variable was attenuated in models including the mediator. Also, these models were adjusted for maternal age and child sex and birth year.

Finally, with Cox regression, we tested if maternal exposure to childhood abuse or neglect, maternal mental and behavioral disorders, and lower maternal education level had additive effects on mental and behavioral disorders in children, adjusting for maternal age and child sex and birth year. We calculated two sum scores: one reflecting the additive effects of maternal exposure to moderate-to-severe levels of childhood abuse (0 = no, 1 = yes) and the other one of maternal exposure to moderate-to-severe levels of childhood neglect (0 = no, 1 = yes) on the top of maternal mental and behavioral disorders (0 = no, 1 = yes), and lower (primary or secondary) education level (0 = no; 1 = yes). The associations between the additive variables and mental and behavioral disorders in children were not time-dependent (*p* values ≥ 0.051).

Using linear regression and structural equation models, we repeated the analyses with psychiatric symptoms in children, assessed with the SDQ total difficulties scale, as the outcome. In these analyses, we adjusted for the same covariates, but used child’s age at the 7–12-year follow-up instead of child’s birth year. Child psychiatric symptoms were examined as continuous square-root-transformed standardized *z* scores.

## Results

Table 1 shows the sample characteristics. Of the mothers, 343 (15.2%) had childhood abuse and 420 (18.7%) childhood neglect scores above the cut-off scores indicating moderate or severe exposure to childhood abuse or neglect, respectively. In our sample, 228 (10.2%) children were diagnosed with mental and behavioral disorders.

**Table 1:** Sample characteristics (*n* = 2252)

Characteristic	Mean (SD)/*n* (%)
Maternal characteristics	
Childhood exposure to emotional, physical, and/or sexual abuse	
Continuous any abuse sum score^a^	18.9 (5.6)
Any abuse above the cut-off indicating moderate-to-severe abuse, yes^b^	343 (15.2%)
Childhood exposure to emotional and/or physical neglect	
Continuous any neglect sum score^c^	16.2 (5.9)
Any neglect above the cut-off indicating moderate-to-severe neglect, yes^d^	420 (18.7%)
Age at delivery, years	32.0 (4.6)
Education level	
Primary or secondary	572 (25.4%)
Tertiary	1680 (74.6%)
Maternal income	
≤ 2000 Euros per month	425 (18.9%)
> 2000 Euros per month	1798 (79.8%)
Data missing	29 (1.3%)
Maternal occupational status	
Employed or self-employed	1928 (85.6%)
Unemployed	50 (2.2%)
Other	233 (10.3%)
Data missing	41 (1.8%)
Lifetime mental and behavioral disorders^e^	
Mental and behavioral disorder by the child’s first mental and behavioral disorder diagnosis or by the end of register follow-up (31/12/2018), yes	308 (13.7%)
Mental and behavioral disorder by the child’s psychiatric symptom assessment, yes	313 (13.9%)
Child characteristics	
Sex, male	1163 (51.6%)
Age at end of register follow-up (31/12/2018), years	10.2 (0.7)
Age at psychiatric symptom assessment, years	9.4 (0.8)
Mental and behavioral disorders, yes^f^	228 (10.1%)
Strength and Difficulties Questionnaire Total difficulties score	5.9 (4.6)

^a^A continuous sum score of maternal exposure to childhood abuse in her own childhood, summing up Childhood Trauma Questionnaire (CTQ) subscale scores on physical, sexual and emotional abuse

^b^A dichotomous cut-off score of maternal exposure to childhood abuse, indicating whether the mother scored above any of the cut-off scores for moderate-to-severe childhood abuse (≥ 10 physical abuse, ≥ 13 emotional abuse, and  ≥ 8 sexual abuse), provided by the CTQ manual. The variable reflects maternal exposure to moderate-to-severe childhood abuse in her own childhood

^c^A continuous sum score of maternal exposure to childhood neglect in her own childhood, calculated by summing up the CTQ scores of subscales on emotional and physical neglect

^d^A dichotomous cut-off score of maternal exposure to childhood neglect. This variable was calculated using the CTQ manual cut-off scores (≥ 10 physical neglect and ≥ 15 emotional neglect). It reflects maternal exposure to any type of moderate-to-severe childhood neglect in her own childhood

^e^Diagnosed with International Classification of Diseases 8th revision (ICD-8) codes 290–315 until 1986; ICD-9 codes 290–319 in 1987–1995; and ICD-10 codes F00–F99 from 1996 onwards

^f^Diagnosed according to ICD-10 codes: F00–F99

Online Supplementary Table S2 shows that maternal exposures to childhood abuse and to childhood neglect were both associated with significantly higher odds of the mother having a mental and behavioral disorder and belonging to the groups with only primary or secondary education level, lower income level, and the ‘other’ occupational status category.

Online Supplementary Table S3 shows that maternal mental and behavioral disorders, and lower maternal education and income levels and maternal unemployment status were associated with higher hazards of mental and behavioral disorders and higher total difficulties scores in children. Boys had higher hazards of mental and behavioral disorders and higher total difficulties scores than girls. Younger maternal age at childbirth was associated with higher total difficulties scores in children (Table S3).

### Maternal exposure to childhood maltreatment and mental and behavioral disorders and psychiatric symptoms in children

Table 2 shows that in models adjusted for child’s sex and birth year or age and maternal age, children of mothers with higher childhood abuse exposure scores and with abuse exposure scores above the cut-off indicating moderate-to-severe exposure to abuse had significantly higher hazards of mental and behavioral disorders and higher total difficulties scores. Maternal exposure to childhood neglect was not associated with mental and behavioral disorders, but it was significantly associated with higher total difficulties scores in children (Table 2).

**Table 2:** Maternal exposure to childhood abuse and neglect and mental and behavioral disorders and total difficulties psychiatric symptoms in children

	Mental and behavioral disorders in children^a^		SDQ total difficulties in children^b^	
	HR (95% CI)	*p*	*B* (95% CI)	*p*
Maternal exposure to childhood abuse continuous sum score^c^				
Model 1	1.20 (1.06–1.37)	0.004	0.20 (0.16–0.24)	< 0.001
Model 2	1.11 (0.98–1.26)	0.11	0.18 (0.14–0.22)	< 0.001
Maternal childhood abuse score above the cutoff indicating exposure to moderate to severe abuse, yes vs. no^d^				
Model 1	1.48 (1.08–2.05)	0.02	0.36 (0.25–0.48)	< 0.001
Model 2	1.22 (0.87–1.70)	0.24	0.29 (0.18–0.40)	< 0.001
Maternal exposure to childhood neglect continuous sum score^e^				
Model 1	1.08 (0.95–1.23)	0.23	0.18 (0.14–0.22)	< 0.001
Model 2	1.00 (0.88–1.14)	0.94	0.16 (0.12–0.20)	< 0.001
Maternal childhood neglect score above the cutoff indicating exposure to moderate to severe neglect, yes vs. no^f^				
Model 1	0.97 (0.70–1.36)	0.86	0.33 (0.22–0.43)	< 0.001
Model 2	0.82 (0.58–1.15)	0.25	0.27 (0.17–0.38)	< 0.001

Model 1 is adjusted for child sex and birth year and maternal age (models with child diagnosis) and child sex and age and maternal age (models with child total difficulties)

Model 2 is further adjusted for maternal lifetime mental and behavioral disorders and maternal education level (all models)

*HR* hazard ratio, *CI* confidence interval, *B* unstandardized regression coefficients from linear regression models, *SDQ* Strengths and Difficulties Questionnaire

^a^Diagnosed according to International Classification of Diseases, 10th revision codes: F00–F99

^b^SDQ total difficulties psychiatric symptom score, expressed in standard deviation units

^c^A continuous sum score of maternal exposure to childhood abuse in her own childhood, summing up Childhood Trauma Questionnaire (CTQ) subscale scores on physical, sexual, and emotional abuse. The sum score is expressed in standard deviation units

^d^A dichotomous cut-off score of maternal exposure to childhood abuse in her own childhood, indicating whether the mother scored above any of the cut-off scores for moderate-to-severe childhood abuse (≥ 10 physical abuse, ≥ 13 emotional abuse, ≥ 8 sexual abuse). The cut-off scores were provided by the CTQ manual. The variable reflects maternal exposure to any type of moderate-to-severe childhood abuse in her own childhood

^e^A continuous sum score of maternal exposure to childhood neglect in her own childhood, calculated by summing up the CTQ scores of the scales on emotional and physical neglect. The sum score is expressed in standard deviation units

^f^A dichotomous cut-off score variable of maternal exposure to childhood neglect in her own childhood. We used the CTQ manual cut-off scores (10 physical neglect and ≥ 15 emotional neglect) to calculate this variable, which reflects maternal exposure to any type of moderate-to-severe childhood neglect in her own childhood

When we adjusted the models showing the associations between maternal exposure to childhood abuse and mental and behavioral disorders in her children for maternal mental and behavioral disorders and maternal education level, the significant associations became non-significant (Table 2). While the associations between maternal exposure to childhood abuse and neglect with higher total difficulties in children remained significant, the effect sizes, as indicated by the regression coefficients, decreased.

### Mediation by maternal mental and behavioral disorders and education level

**Fig. 1:**
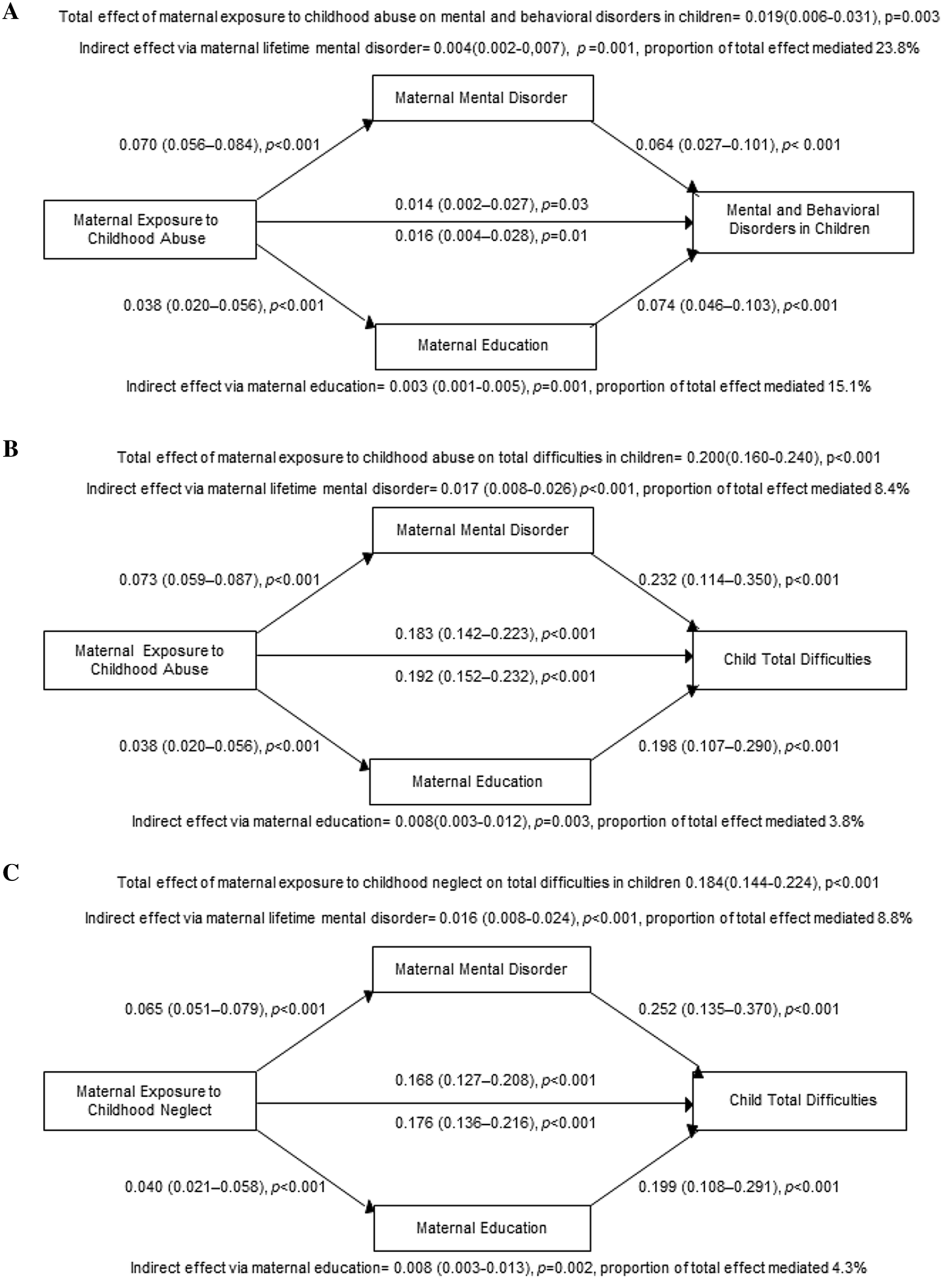
Maternal lifetime mental and behavioral disorders and education level partially mediate the association between maternal exposure to childhood abuse and mental and behavioral disorders in children (panel **a**). They also partially mediate the associations of maternal exposure to childhood abuse (panel **b**) and childhood neglect (panel **c**) with child total difficulties, captured with the Strengths and Difficulties Questionnaire. Mediation analyses were conducted with structured equation models and in separate models for the mediators. The models are adjusted for maternal age and child’s sex and birth year (panel **a**) and maternal age and child’s sex and age at psychiatric symptom assessment (panel **b**, **c**). Numbers represent unstandardized regression coefficients, 95% confidence intervals, and *p* values. Maternal abuse and neglect exposure continuous sum scores and child total difficulties scores are expressed in standard deviation units

Figure 1 shows that maternal mental and behavioral disorders and education level partially mediated the effect of maternal exposure to childhood abuse on mental and behavioral disorders in children. Figure 1 also shows that maternal mental and behavioral disorders and education level partially mediated the effects of maternal exposure to childhood abuse and neglect on total difficulties in children.

### Additive effects of maternal exposure to childhood maltreatment, maternal mental and behavioral disorders, and lower education level

**Fig. 2:**
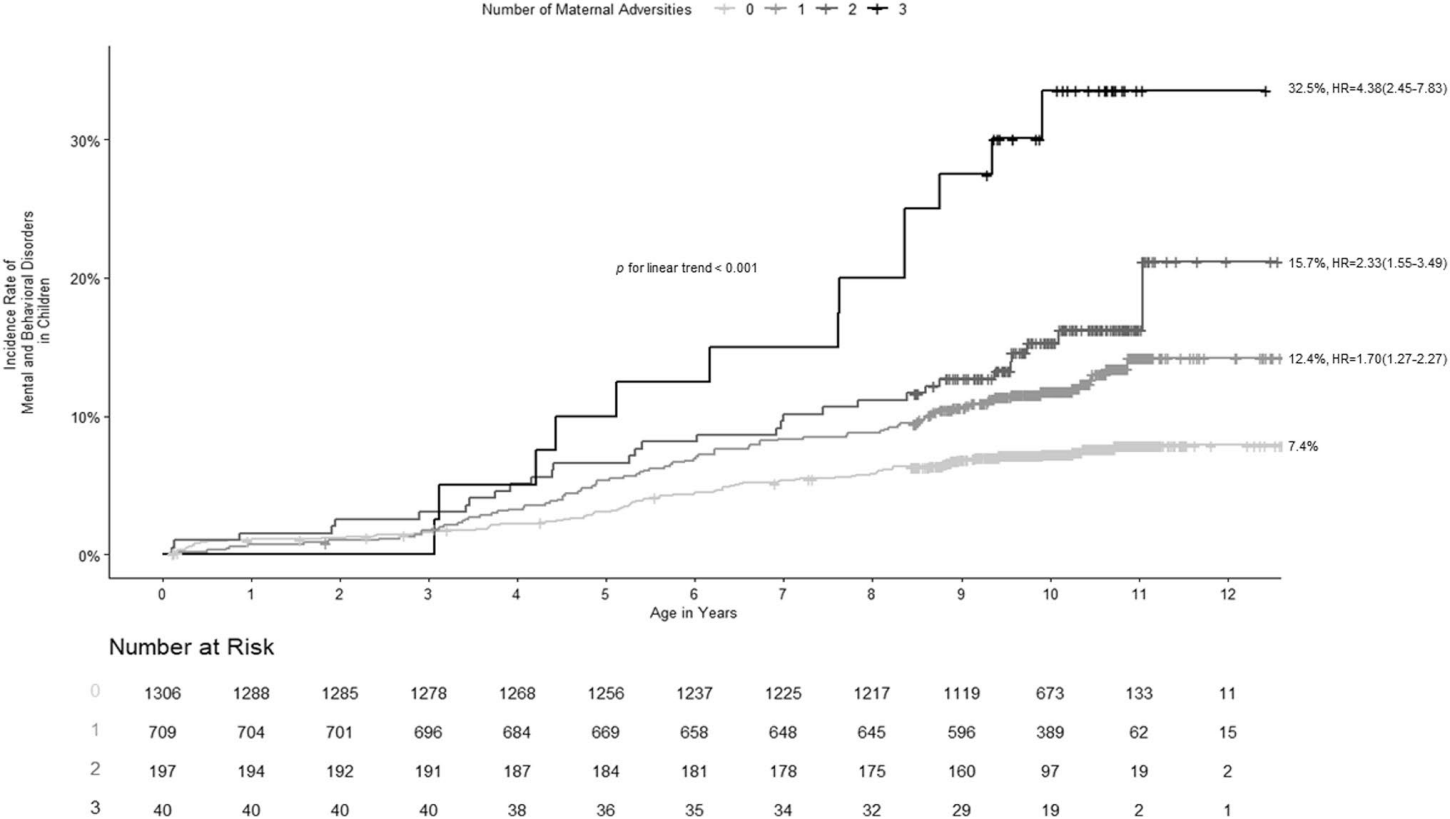
The incidence rates and cumulative incidences of mental and behavioral disorders in children whose mothers have none, one, two, or three of the maternal adversities, namely maternal exposure to moderate-to-severe childhood abuse, maternal lifetime mental and behavioral disorders, and/or lower education level. The figure shows the additive effects of maternal exposure to these adversities on mental and behavioral disorders in children. The curves show incidence rates and the numbers represent cumulative incidences of mental and behavioral disorders in children, hazard ratios (HR), and 95% confidence intervals (CI); *p* values refer to linear trends from Cox models adjusted for maternal age and child’s sex and birth year

Figure 2 shows that maternal exposure to childhood abuse, maternal mental and behavioral disorders, and lower maternal education level had additive effects increasing the hazards of mental and behavioral disorders in children, adjusting for child sex and birth year and maternal age. The cumulative incidences of mental and behavioral disorders in children were 7.4%, 12.4%, 15.7%, and 32.5% according to the number of adversities the mother was exposed to (from 0 to 3, respectively).

**Fig. 3:**
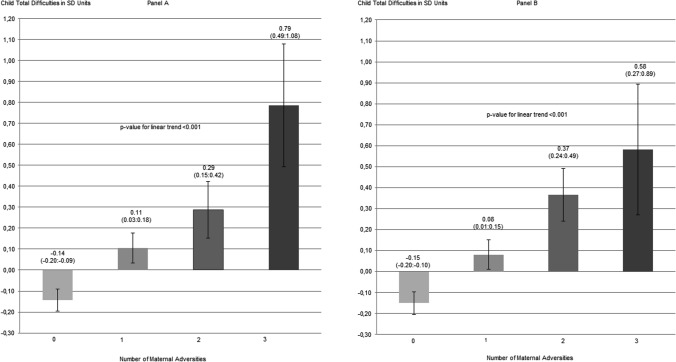
Total difficulties psychiatric symptom scores, captured by the Strength and Difficulties Questionnaire, in children whose mothers have none, one, two, or three of the maternal adversities. These adversities included maternal exposure to moderate-to-severe childhood abuse, maternal lifetime mental and behavioral disorders, and/or lower education level (panel **a**) and maternal exposure to moderate-to-severe childhood neglect, maternal lifetime mental and behavioral disorders, and/or lower education level (panel **b**). The figure shows estimated marginal means and 95% confidence intervals of child total difficulties according to number of maternal adversities, adjusted for maternal age and child’s sex and age. Child total difficulties scores are expressed in standard deviation units; *p* values refer to linear trends from linear regression models

Figure 3 shows that total difficulties’ scores in children also increased according to the number of adversities the mother was exposed to. This was true both when the adversities sum score included maternal exposure to childhood abuse and when it included maternal exposure to childhood neglect.

## Discussion

In a large cohort of Finnish mothers and children, we showed that children of mothers who themselves had been exposed to childhood abuse had 1.2 times higher hazards of any mental and behavioral disorder from birth to 8.4–12.8 years. This association became non-significant after adjustments for maternal mental and behavioral disorders and education level, which suggested that these variables might mediate the association between maternal exposure to childhood abuse and mental and behavioral disorders in children. Indeed, mediation analyses corroborated partial mediation via maternal mental and behavioral disorders and education level, with the proportion of effect size mediated being over 15% for both mediators. We also showed that together with maternal exposure to childhood abuse, maternal mental and behavioral disorders, and lower maternal education level added to the hazard of mental and behavioral disorders in children. The cumulative incidence of mental and behavioral disorders was the highest for children whose mothers were exposed to all three adversities (32.5% vs. 7.4% for children of mothers exposed to none of the adversities). We showed similar associations between maternal exposure to childhood abuse and child psychiatric symptoms as indexed by mother-rated child total difficulties. Child total difficulties were also associated with maternal exposure to childhood neglect.

Our findings support the previous research literature which has shown that maternal exposure to childhood maltreatment is associated with mental health adversities captured by psychiatric symptom scales in children [12–18]. They also support the findings of two small studies in one Australian cohort demonstrating an association between maternal exposure to childhood maltreatment and diagnosed emotional disorders in children at the age of 4 years [20, 21]. Our study suggests that the association may extend to any mental and behavioral disorder in children as an outcome phenotype. While we also showed that both maternal exposure to childhood abuse and childhood neglect were associated with increased psychiatric symptoms in children, our study suggested that the intergenerational transmission into diagnosed mental and behavioral disorders in children may be more specific to maternal exposure to childhood abuse. This may reflect that childhood abuse may be a more severe form of maltreatment than neglect, and/or that diagnosed disorders as outcomes may capture more severe symptomatology than psychiatric symptom scales. Thus, our findings add significantly to the scarce literature by showing that the intergenerational transmission of the effects of maternal exposure to childhood abuse extend to more severe, objectively defined diagnosed mental and behavioral disorders in children. Our findings also suggest that the associations between maternal exposure to childhood maltreatment and mother-rated psychiatric symptoms in her children are, at least fully, not explained by rater bias or shared method variance inflating the mother–child associations. In regression models adjusting for maternal education and mental and behavioral disorders, maternal exposure to childhood maltreatment was, however, independently associated with psychiatric symptoms but not with mental and behavioral disorders in children. This may indicate that familial pathways would more strongly mediate the intergenerational associations with diagnosed mental and behavioral disorders in children. However, psychiatric symptoms and mental and behavioral disorders in children were strongly associated with each other in our study sample (odds ratio for mental and behavioral disorders per each standard deviation unit increase in psychiatric symptoms in children = 3.09, 95% confidence interval = 2.63–3.63, *p* < 0.001) and mediation via maternal mental and behavioral disorders and education level was partial for both child mental health phenotypes. Hence, the above-mentioned difference may be due to the higher statistical power present for the analysis of continuous than of categorical outcomes.

In keeping with the previous literature, which has demonstrated that maternal self-reported psychiatric symptoms [13, 15, 18] and maternal physician-diagnosed major depressive disorder [16] at least partially mediate the associations between maternal exposure to childhood maltreatment and mother-rated psychiatric symptoms in children, our study showed that the same holds true for maternal and child mental and behavioral disorder diagnoses. While education level was mother-reported in our study, it also partially mediated the link between maternal exposure to childhood abuse and mental and behavioral disorders in children. We found similar mediation effects via maternal mental and behavioral disorders and education also on mother-reported psychiatric symptoms in children. Maternal mental and behavioral disorders and lower education may thus be among the mediating pathways of intergenerational transmission of maternal exposure to childhood maltreatment to mental and behavioral disorders and psychiatric symptoms in children.

However, while the mediation by maternal mental disorders was 24% and by maternal education level it was 15%, a significant proportion of the effect was not mediated by them. Other possible mediating pathways include postnatal environmental pathways, including insecure attachment, suboptimal parenting, and the recurrence of maltreatment of the child. Previous studies have shown that children of parents exposed to childhood maltreatment are more likely exposed to childhood maltreatment themselves [15, 17, 33, 34], and that exposure to childhood maltreatment is associated with more maladaptive and insensitive parenting and insecure attachment in adulthood [13, 14, 33]. Indeed, maternal insecure attachment [14] and intergenerational recurrence of the maltreatment of the child [15, 17] have been shown to partially mediate the associations of maternal exposure to childhood maltreatment in her own childhood with psychiatric symptoms in her children. Also, insensitive parenting is associated with psychiatric problems in children [15, 16]. However, findings on these mediation pathways are inconclusive.

Although many studies have proposed such postnatal environmental mechanisms as primary mediating pathways, previous research also suggests that the intergenerational transmission of maternal exposure to childhood maltreatment to offspring mental disorders may begin during fetal life [7, 13–15], which is a sensitive period for the structural and functional development of the brain [7, 35]. Thus, among the possible pathways are also changes in gestational biology resulting from alterations in endocrine and immune-inflammatory stress physiology, including hypothalamic–pituitary–adrenal axis dysregulation, higher cortisol levels, and increased placental corticotrophin-releasing hormone concentrations [7], increasing later risk of mental disorders in children. In addition, mothers with childhood maltreatment history have higher risks of suffering from psychological distress (depressive or anxiety symptoms) during pregnancy [5, 15]. Maternal psychological distress during pregnancy, in turn, is consistently associated with increased risks of psychiatric symptoms and mental disorders in children [15, 35–37]. Hence, further studies are warranted to identify additional mediators not captured here. These may include the above-mentioned postnatal environmental pathways, along with maternal psychosocial factors, such as intimate partner violence [13, 18], stressful life events [13, 17], unemployment [23, 24], lack of social support [15, 17], each of which are associated with maternal exposure to childhood maltreatment and with psychiatric symptoms in children [17, 18], and biological mediators, such as child hypothalamic–pituitary–adrenal axis functioning, low-grade inflammation, or epigenetic modifications with alterations programmed already in utero or early childhood [7, 38].

Furthermore, exposure to maltreatment as a child may result in the accumulation and circular associations of the adversities [12–15, 33], and thereby explain why the associations of maternal exposure to childhood maltreatment with mental and behavioral disorders in her children may not be independent of these maternal adversities and why they may mediate and add to the effects. While maternal mental and behavioral disorders and education level acted here as mediators, they also added to the adverse effects of maternal exposure to childhood abuse on mental and behavioral disorders and maternal exposure to childhood abuse and neglect on psychiatric symptoms in children. Our findings showed that the hazard of mental and behavioral disorders and psychiatric symptoms increased linearly according to the number of maternal adversities. These findings together with the mediation findings could be seen as indicating that shared familial risk factors (as indicated by the familial risk for psychopathology and lower maternal education) need to be in place for maternal exposure to childhood maltreatment to have intergenerational effects on offspring mental disorders. However, exposure to childhood maltreatment may be a causal risk factor for mental disorders within the same generation [3, 4, 11], and prospectively predicts an increased risk of adulthood socioeconomic adversities independently of childhood socioeconomic adversities [24]. Hence, the findings rather indicate maternal mental and behavioral disorders and socioeconomic adversity as intermediate, mediating, and additively contributing factors within the intergenerational pathway from maternal exposure to childhood maltreatment in her own childhood to mental health problems in her children.

The strengths of our study include a large sample size, the assessment of maternal exposure to childhood maltreatment with a validated questionnaire, and the prospective assessment of physician-diagnosed mental and behavioral disorders in children. Instead of focusing on maternal exposure to any type of childhood maltreatment, we examined maternal exposure to childhood abuse and childhood neglect separately. Furthermore, we focused on all mental and behavioral disorder diagnoses in the children until ages 8.4–12.8 years, identified from the validated [29–31] HILMO register. This decreases rater bias and shared method variance present in studies where both predictor and outcome phenotypes are assessed by the mother [19]. We were also able to identify lifetime register-based, validated [29] physician-diagnoses of mental and behavioral disorders for the mothers. Still, we also examined psychiatric symptoms in children, assessed with a validated [32] questionnaire, thereby consolidating previous findings.

Our study also has limitations. Compared to the non-participants of the original PREDO cohort, the mothers and children of our study sample less frequently had diagnosed mental and behavioral disorders. The participating mothers also had higher education levels. Hence, selective follow-up attrition cannot be disregarded. Only half of the original cohort participated in the follow-up, which limits the external validity of our findings. Our sample comprised mother–child dyads from a Nordic, high-resource setting. Although the homogeneity of our sample is a strength, it also limits the generalizability of our findings to other populations. Another study limitation is the retrospective self-report assessment of maternal exposure to childhood maltreatment. Meta-analysis shows that retrospective and prospective assessments of childhood maltreatment show relatively low agreement [39]. Many factors may contribute to this. Among them are memory biases in retrospective assessments leading to both false-positive and false-negative assessments [4, 17, 39]. However, the sensitivity of prospective assessments may also be low, since only the most severe cases may gain the attention of authorities and thus be registered in child welfare registers which are often used as the data sources for prospective studies [4, 17, 39]. Nevertheless, retrospectively and prospectively ascertained exposure to childhood maltreatment do show similar associations with mental disorders in the same generation [4, 6, 8]. Retrospective and prospective assessment methods of childhood maltreatment exposure can thus be considered complementary. Future studies would benefit from using both assessment methods also when examining intergenerational consequences.

We are among the first to show intergenerational associations of maternal exposure to childhood abuse to diagnosed mental and behavioral disorders in her children. Our findings highlight the role of maternal lifetime mental disorders and education level as mediating pathways of this intergenerational transmission, and as factors that may additively with maternal exposure to childhood abuse accumulate the child’s risk. This finding of additive effects is novel and may allow identifying at-risk children for timely targeted prevention interventions years before the occurrence of clinically manifest symptoms. Overall, our findings highlight the importance of targeted personalized preventive measures to mothers exposed to childhood maltreatment and their children to prevent intergenerational transmission of risk [13].

In conclusion, our large study showed that maternal exposure to childhood abuse but not to childhood neglect is associated with increased hazards of mental and behavioral disorders in her children, while both maternal exposure to childhood abuse and to childhood neglect are associated with increased psychiatric symptoms in children. These associations are partially mediated by maternal lifetime mental and behavioral disorders and education level. The hazards of mental disorders are the highest for the children of mothers with exposure to childhood abuse history, mental and behavioral disorders, and lower education level. Interventions for these mothers have the potential to prevent the intergenerational transmission of mental disorders in their children.

### Supplementary Information

Below is the link to the electronic supplementary material.

Supplementary file1 (PDF 153 KB)

## Data Availability

The code for the analysis is available on request from the corresponding author (marius.lahti-pulkkinen@helsinki.fi).

## Data availability

Due to its sensitive nature, we cannot make the study data open access. Requests to access the dataset from qualified researchers trained in confidentiality protocols can be sent to PREDO study board. Data requests will require further review by ethical committees and register authorities.
