# Supplementary material for: Maternal exposure to childhood maltreatment and mental and behavioral disorders in children
Source: Eur Child Adolesc Psychiatry. 2022 Oct 1;32(12):2463–75. doi: 10.1007/s00787-022-02090-8 (PMC10682113; doi:10.1007/s00787-022-02090-8)
Supplement: Supplementary file 1 — Supplementary file1 (PDF 153 KB) [file 787_2022_2090_MOESM1_ESM.pdf]

Maternal Exposure to Childhood Maltreatment and Mental and Behavioral Disorders in Children.

European Child & Adolescent Psychiatry. Authors: Aino Airikka, Marius Lahti-Pulkkinen, Soile Tuovinen, Kati Heinonen, Jari Lahti, Polina Girchenko, Anna Lähdepuro, Riikka Pyhälä, Darina Czamara, Pia Villa, Hannele Laivuori, Eero Kajantie, Elisabeth B. Binder, Katri Räikkönen. Corresponding Author: Marius Lahti-Pulkkinen. Affiliations: Department of Psychology and Logopedics, Faculty of Medicine, University of Helsinki, Helsinki, Finland; Finnish Institute for Health and Welfare, Helsinki, Finland; University of Edinburgh, Edinburgh, United Kingdom. E-mail: marius.lahti-pulkkinen@helsinki.fi.

Supplementary Table S1. The associations between maternal education, occupation and income. Percentage of women in different categories and p-values from χ^2-tests for the associations between the different indicators of socioeconomic status.

Maternal Socioecomic Indicator	Maternal Income Level per Month			Maternal Occupational Status			
	≤2000 euros	>2000 Euros		Unemployed	Employed or Self-Employed	Other[a]	
	n(%)	n(%)	p	n(%)	n(%)	n(%)	p
Maternal Education Level							
Primary or Secondary	202(35.9%)	361(64.1%)	<0.001	21(3.8%)	439(78.5%)	99(17.7%)	<0.001
Tertiary	223(13.4%)	1437(86.6%)		29(1.8%)	1489(90.1%)	134(8.1%)	
Maternal Occupational Status							
Unemployed	41(83.7%)	8(16.3%)	<0.001	-			
Employed or Self-Employed	186(9.7%)	1723(90.3%)					
Other[a]	175(77.8%)	50(22.2%)					

[a] This category included women who reported their occupational status as currently studying, retired, staying home with children or other than any of the other occupational statuses listed here.

Supplementary Table 2. The Univariate association of Maternal Exposure to Childhood Abuse and Childhood Neglect with Maternal Mental and Behavioral Disorders, Education Level, Income Level and Occupation Status in Adulthood. Odds Ratios and 95% Confidence Intervals from Logistic Regression Models.

Maternal Phenotype in Adulthood	Maternal Mental and Behavioral Disorders[a]		Maternal Education Level (Primary or Secondary vs. Tertiary)		Maternal Income Level (≤2000 Euros vs. >2000 Euros)		Maternal Occupational status: Unemployed vs. Self-employed/Employed		Maternal Occupational status: Other vs. Self-employed/Employed	
	OR (95% CI)[b]	p	OR (95% CI)[b]	p	OR (95% CI)[b]	p	OR (95% CI)[b]	p	OR (95% CI)[b]	p
Maternal exposure to childhood abuse										
Continuous sum score[c]	1.79(1.59-2.01)	<0.001	1.22(1.11-1.34)	<0.001	1.30(1.17-1.44)	<0.001	1.37(1.05-1.80)	0.02	1.32(1.16-1.51)	<0.001
Score above the cutoff indicating exposure to moderate to severe abuse, yes vs. no[d]	3.31(2.53-4.34)	<0.001	1.73(1.36-2.22)	<0.001	1.47(1.12-1.93)	0.01	1.73(0.88-3.42)	0.11	1.59(1.13-2.24)	0.01
Maternal Exposure to Childhood Neglect										
Continuous sum score[e]	1.73(1.53-1.95)	<0.001	1.23(1.12-1.35)	<0.001	1.24(1.12-1.38)	<0.001	1.24(0.94-1.65)	0.13	1.13(0.99-1.30)	0.07
Score above the cutoff indicating exposure to moderate to severe abuse, yes vs. no[f]	2.76(2.13-3.58)	<0.001	1.39(1.10-1.76)	0.01	1.52(1.18-1.96)	0.001	1.02(0.49-2.12)	0.95	1.51(1.09-2.08)	0.01

[a] International Classification of Diseases 8th revision (ICD-8) codes 290-315 until 1986; ICD-9 codes 290-319 in 1987-1995; and ICD-10 codes F00-F99 from 1996 onwards. All diagnoses until 31/12/2018 used for the purposes of this table.

[b] OR=Odds ratio; 95% CI= 95% Confidence Interval from logistic regression analyses.

[c] A continuous sum score of maternal exposure to childhood abuse in her own childhood, summing up the Childhood Trauma Questionnaire (CTQ) subscale scores on physical, sexual and emotional abuse.

[d] A dichotomous cutoff score of maternal exposure to childhood abuse in her own childhood, indicating whether the mother scored above any of the cutoff scores for moderate to severe childhood abuse (≥10 physical abuse, ≥13 emotional abuse, ≥8 sexual abuse), provided by the CTQ manual. The variable reflects maternal exposure to any type of moderate to severe childhood abuse in her own childhood.

[e] A continuous sum score of maternal exposure to childhood neglect in her own childhood, calculated by summing up the CTQ scores of the scales on emotional and physical neglect.

[f] A dichotomous cutoff score of maternal exposure to childhood neglect in her own childhood. We used the CTQ manual cutoff scores (10 physical neglect and ≥15 emotional neglect) to calculate a variable, which reflects maternal exposure to any type of moderate to severe childhood neglect in her own childhood.

SUPPLEMENTARY TABLE S3. The Associations of Covariates with Mental and Behavioral Disorders and Psychiatric Symptoms in Children.

Characteristics	Mental and Behavioral Disorders[a]		SDQ Total Difficulties[b]	
	***HR* (95 % CI)**	***p***	***B* (95 % CI)[b]**	***p***
**Maternal Characteristics**				
Age at delivery[b]	1.02 (0.89–1.16)	0.78	-0.05 (-0.09-(-0.01))	0.03
Mental and behavioral disorder[c] (yes/no)	1.85 (1.35–2.53)	<0.001	0.37 (0.25–0.49)	<0.001
Education (primary or secondary/ tertiary)	2.01 (1.54–2.62)	<0.001	0.24 (0.14–0.33)	<0.001
Income (≤ 2000 Euros vs. >2000 Euros per month)	1.46 (1.08-1.97)	0.01	0.18(0.07-0.28)	0.001
Occupational Status:				
Unemployed vs. Employed/Self-Employed	2.22(1.18-4.20)	0.01	0.32(0.04-0.59)	0.02
Other vs. Employed/Self-Employed	1.40(0.95-2.06)	0.09	0.10(-0.03-0.24)	0.14
**Child Characteristics**				
Sex (male/female)	2.04 (1.55–2.70)	<0.001	0.31 (0.23-0.39)	0.001
Birth year/Child age at psychiatric symptom assessment[b]	0.97 (0.85–1.11)	0.70	-0.02 (-0.06–0.03)	0.46

Note: HR=Hazard Ratio; B=Unstandardized Regression Coefficients; CI=Confidence Interval; SDQ = Strengths and Difficulties Questionnaire

[a]Diagnosed according to the International Statistical Classification of Diseases and Related Health Problems (ICD) 10th revision codes: Mental and behavioral disorders (F00-F99)

[b]Scores are expressed in standard deviation units.

[c]Maternal lifetime mental and behavioral disorder diagnosis (Diagnosed according to ICD-8 codes 290-315 until 1986; ICD-9 codes 290-319 in 1987-1995; ICD-10 codes F00-F99 from 1996 onwards) by child's first mental disorder diagnosis (models with child diagnosis) or by follow-up (models with child psychiatric symptoms).
